# University Students’ Perceptions and Intentions to Use Digital Mental Health Services Including Online Therapy and Mental Health Apps: A Cross-Sectional Study

**DOI:** 10.3390/ijerph23060719

**Published:** 2026-05-28

**Authors:** Tamadhir Al-Mahrouqi, Maryam Al Wardy, Abdullah Al Lawati, Ahmed Al Maskari, Alazhar Al Azri, Qaiser Al Riyami, Hamood Al Aufi, Sachin Jose, Hamed Al Sinawi

**Affiliations:** 1 Department of Behavioral Medicine, College of Medicine and Health Sciences, Sultan Qaboos University, Muscat 123, Oman; t.almahrouqi@squ.edu.om; 2 Department of Behavioral Medicine, Sultan Qaboos University Hospital, Muscat 123, Oman; abdlawati1@gmail.com (A.A.L.);; 3 Department of Clinical Biochemistry, Sultan Qaboos University Hospital, Muscat 123, Oman; al.alazri@squ.edu.om; 4 Research Section, Oman Medical Specialty Board, Muscat 123, Oman; sachin.j@omsb.org

**Keywords:** digital mental health, telepsychiatry, mental health apps, online therapy

## Abstract

**Highlights:**

**Public health relevance—How does this work relate to a public health issue?**

- The increase in mental health difficulties among young adults poses a burden on traditional mental health services in Oman, and digital mental health tools could offer scalable solutions to improve access to public health care.
- Digital mental health tools are favored for their ease of access and 24/7 availability, yet concerns remain about their cultural, legal, and religious relevance.

**Public health significance—Why is this work of significance to public health?**

- This study explores the predictive relationship between attitudes and beliefs about digital mental health tools and the intention to use such tools, with perceptions of online therapy and mental health apps as potential mediators of this relationship.
- Positive attitudes toward digital technology are associated with greater intention to use these tools, underscoring the need for extensive psychoeducation to promote digital mental health solutions as a resource before their implementation in healthcare institutions.

**Public health implications—What are the key implications or messages for practitioners, policy makers and/or researchers in public health?**

- Only mental health apps positively mediated the relationship between attitudes and intention, highlighting the importance of service-level perceptions in shaping behavioral intentions to use digital mental health services.
- Adoption of digital mental health services could be influenced by how such services are evaluated within their social context, as mental health apps offer greater privacy and accessibility than online therapy sessions.

**Abstract:**

This study examined the association between attitudes toward digital technology and intentions to use digital mental health solutions, with perceptions of mental health apps and online therapy as potential mediators. A cross-sectional survey was conducted with 360 undergraduate students at Sultan Qaboos University (SQU) (July 2025). The mean age was 21.24 (SD = 3.12), with 50.3% of female respondents and 49.7% male. Using a validated questionnaire, we included assessments of attitudes and beliefs about digital technology, perceptions of mental health apps and online therapy, and intention to use digital mental health solutions. Mediation analysis was performed using structural equation modeling. Attitudes toward digital technology were significantly associated with intention (β = 0.358, *p* < 0.001). Perceptions of mental health apps significantly mediated this relationship (indirect effect = 0.065, *p* = 0.006), while perceptions of online therapy did not (indirect effect = 0.030, *p* = 0.109). The total indirect effect through both mediators was significant (0.095, *p* = 0.002), with a strong total effect on intention (0.454, *p* < 0.001). These findings suggest that improving students’ perceptions of mental health apps may help them use digital mental health services. Implementation strategies in similar university settings should target service-specific perceptions to drive adoption of these tools.

## 1. Introduction

Globally, nearly one in eight people, about 970 million, were suffering from a mental health disorder in 2019, with predicted global rises of 26% in anxiety and 28% in depression post COVID-19 [1,2]. Such findings have been reported among undergraduate college students [3]. Many students live away from home for the first time during emerging adulthood and often experience significant changes in academic, financial, and social pressures. Findings supporting this perspective were demonstrated by Ibrahim et al., revealing that 37% of the students had symptoms that scored above moderate depression [4]. These hidden challenges, therefore, pose a significant risk to students’ well-being.

There is no doubt that such challenges are also evident in Oman, as 30% of over 1300 people reported anxiety or depression during the COVID-19 pandemic [5]. Such challenges were also highlighted in studies exploring perceived stress in medical students, with a prevalence of 54% [6]. More recent studies have also emphasized this burden, with a 33.7% prevalence rate of depressive symptoms in Omani undergraduate students in Sultan Qaboos University (SQU) [7]. These findings underscore a growing mental health concern in Oman, mirroring global statistics of increased mental health issues among youth.

These rising trends of depression and anxiety symptoms place increasing pressure on university counseling services. Globally, many student counseling services are understaffed, and the staff involved face occupational burnout [8]. This has led to the utilization of innovative approaches to bridge gaps in mental health care, with Artificial Intelligence (AI) emerging as a promising tool [9]. AI tools stand out for their scalability and ability to deliver real-time, low-threshold support, making them particularly relevant for young populations such as students. The use of AI in mental health covers a wide range of digital services. For example, digital mental health apps often offer accessible support through self-help strategies, particularly in mental health crisis assistance. These features include mood tracking, symptom monitoring, and personalized coping strategies, which are available 24/7 to the user. Subsequently, AI chatbots can also be integrated in mental health care, particularly because they are powered by natural language processing, offering one-to-one support that is interactive and simulates a human dialog in real time [10].

For example, a study by Gbollie et al. [11] found that 60% of students intended to use digital mental health tools for future psychological difficulties. More recent trials have also shown that interactive AI chatbots may reduce depressive and anxiety symptoms more effectively than standardized self-help e-books [12,13]. Participants who interacted with the chatbot showed higher engagement and overall satisfaction than those in the control group. These findings are consistent with other studies and reflect the promising efficacy of digital mental health apps and AI chatbots in mental health support [14].

Although digital mental health tools are increasingly used globally, culturally relevant policies for their implementation in the MENA region remain limited. Existing studies suggest that users value AI tools for their anonymity and constant accessibility, but concerns about security, religious and cultural appropriateness, and mental health stigma remain important barriers [15,16]. This largely corresponds to the barriers associated with their collectivist nature, in which concerns about personal safety are bound to social roles and expectations.

These findings have also been supported in the Omani population. For example, Al-Maskari et al. and Al-Lawati et al. qualitatively and quantitatively explored SQU students’ perceptions, attitudes, and previous experiences towards AI-powered digital mental health tools [16,17]. Their findings showed that participants valued the 24/7 accessibility and anonymity of the digital mental health tools, which made them more approachable than traditional therapy. However, some participants raised concerns about security and legal issues, highlighting the importance of privacy protection in the use of digital mental health tools [18]. Furthermore, the idea that the adopted tools did not accommodate the religious and cultural formalities of Omani society remained a concern for AI users, reflecting their desire for personal meaning in their use of mental health treatment.

These inconsistencies between high digital literacy and low utilization of digital mental health tools suggest that access alone does not fully drive adoption. Rather, there may be mediating psychological or perceptual factors (e.g., beliefs) that predict the extent to which students intend to use digital apps for mental health. Theoretically, beliefs that translate into attitudes towards performing a certain behavior are thoroughly explained by the Theory of Planned Behavior (TPB), suggesting that individuals are more likely to perform a behavior if they have favorable perceptions towards the behavior [19]. Thus, there remains, yet a gap in understanding the relationship between the attitudes, perceptions, and beliefs in driving students’ intended use of digital mental health apps, particularly in regions driven by social harmony. By further evaluating the predictive relationships among these factors, the implementation of a culturally accurate digital mental health tool will be more easily facilitated in collectivist societies such as Oman and the MENA region.

### 1.1. Hypothesis

**Hypothesis** **1:** *More positive attitudes and beliefs about digital technology will be associated with greater intention to use digital mental health solutions.*

**Hypothesis** **2:** *The relationship between attitudes and beliefs about digital technology and intention to use digital mental health solutions will be mediated by perceptions and beliefs about specific digital mental health modalities, namely mental health apps and online therapy.*

### 1.2. Study Objectives

This study aims to explore the predictive relationships among intention to use digital mental health solutions, attitudes toward digital technology, perceptions of mental health apps, and beliefs about online therapy among undergraduate students at Sultan Qaboos University, Sultanate of Oman. The research questions were addressed as follows:

1. Are positive attitudes and beliefs about digital technology associated with greater intention to use digital mental health solutions among university students in Oman?
2. Do perceptions and beliefs about mental health apps and online therapy mediate the relationship between attitudes and beliefs about digital technology and intention to use digital mental health solutions?

## 2. Methods

### 2.1. Study Design and Setting

This cross-sectional study was conducted at Sultan Qaboos University (SQU), the largest governmental university in Oman. SQU includes more than 12 colleges and enrolls a large undergraduate student population, making it a suitable setting for examining students’ views on AI-based mental health tools. Data were collected between 1 July and 31 July 2025.

### 2.2. Inclusion and Exclusion Criteria

All undergraduate students who were actively enrolled at SQU during the 2025–2026 academic year, regardless of nationality, were eligible to participate if they provided informed consent. Postgraduate students, elective students who were not officially enrolled at SQU during the study period, and incomplete or duplicate survey responses were excluded from the final analysis.

### 2.3. Data Collection

Participants completed an online survey via Google Forms. They gave their consent electronically before starting the questionnaire. Students were invited to join the study via the university’s official email system, with three reminder emails sent 10 days apart. The survey link was also shared on the university’s official social media platforms, including its WhatsApp channel, Twitter, and Instagram, to reach as many students as possible.

### 2.4. Outcome Measures

The questionnaire was adapted from Gbollie et al. [11], which was originally developed in English. The questionnaire was administered in English because English is the language of instruction in the university setting where the study was conducted. Therefore, no translation or back-translation procedure was performed. The original response-scale structure was retained to preserve comparability with the source instrument; accordingly, different sections used different Likert-type response formats, including 4-point and 5-point scales, reflecting the structure of the original questionnaire.

### 2.5. Sociodemographic Questionnaire

Participants were asked to provide their age, gender, and whether they were Omani or non-Omani residents living in Oman.

### 2.6. Students’ Attitudes and Intentions Toward AI-Based Mental Health Tools

The questionnaire was adapted from the validated instrument developed by Gbollie et al. [11]. The questions measuring students’ attitudes and intentions toward AI-based mental health tools were drawn from established, validated frameworks used in university settings. In the original study, an exploratory factor analysis revealed three main components: usefulness and safety; perceived effectiveness and trust in AI; and support for data protection. Each component demonstrated strong reliability (Cronbach’s α > 0.80), supporting the tool’s accuracy and consistency. The validated questionnaire included several main sections:

### 2.7. Intention to Use Traditional and Digital Mental Health Services

Participants rated their likelihood of using in-person therapy and various digital mental health options, including internet searches, video-based online therapy, mobile applications, and AI chatbots, on a 5-point Likert scale ranging from 1 (not at all likely) to 5 (very likely).

### 2.8. Perceptions of Digital Technology and Attitudes Toward It

Participants indicated their level of agreement with statements assessing perceived privacy, convenience, effectiveness, accessibility, anonymity, and ease of use of AI-powered mental health tools, as well as attitudes toward AI and online privacy.

### 2.9. Perceptions of Mental Health Apps

Participants rated the importance of selected mental health app features, including privacy, data protection, and local development, on a 4-point scale (See Supplementary Table S1), as well as their preferred focus areas and priorities in mental health app features (See Supplementary Table S2).

### 2.10. Perceptions of Online Therapy

Participants answered several five-point scale questions about online therapy, including whether they thought it was convenient, private, and effective.

### 2.11. Sample Size Calculation

The minimum sample size was calculated using the standard formula for population proportions, assuming a 95% confidence level, a 5% margin of error, and an expected proportion of 30% based on anticipated awareness and use of AI-based mental health tools among university students. This produced an initial estimate of 323 participants. After applying the finite population correction to the total number of undergraduate students at SQU in 2024–2025 (about 15,228), the final required sample size was approximately 360 students.

### 2.12. Statistical Analysis

**Figure 1:**
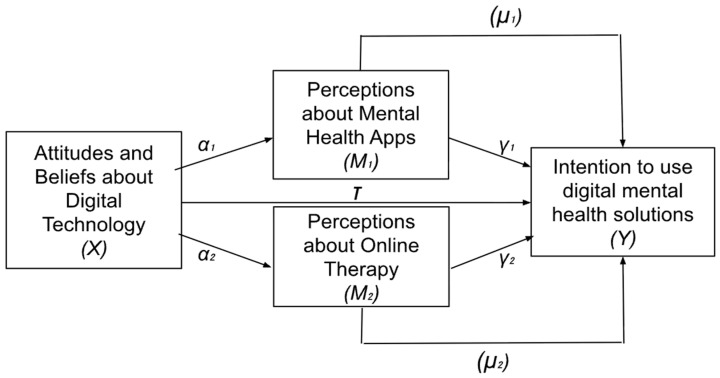
A hypothesized conceptual model for the mediational roles of perception about mental health apps (*M*_1_/*μ*_1_) and online therapy (*M*_2_/*μ*_2_) in the relationship between Attitudes about digital technology (*X*) and intention to use digital mental health solutions (*Y*).

Statistical analyses were conducted using Jamovi version 2.6.44 [20]. Descriptive statistics, including frequencies, percentages, means, standard deviations (SD), medians, and ranges, were used to summarize the students’ demographic and psychological characteristics. In the hypothesized conceptual model (Figure 1), the total association between attitudes and beliefs about digital technology (X1) and intention to use digital mental health solutions (Y) was considered. This total association comprised a direct pathway between X1 and Y and an indirect pathway via perceptions of mental health apps (M1) and perceptions of online therapy (M2). Direct and indirect associations were examined using mediation analysis. Standardized coefficients (β), standard errors (SE), z-statistics, and *p* values were reported for each pathway. Model fit was assessed using R^2^ (coefficient of determination). The full information maximum likelihood (FIML) approach was used for model estimation.

## 3. Results

### Descriptive Statistics

A total of 360 students participated in the study (Table 1). The sample was nearly equally split between males (49.7%) and females (50.3%), with a mean age of 21.24 ± 3.12 years. The majority were Omani (95%) and identified as students (98.6%). Most reported strong digital literacy, with over half being fairly or very confident using digital tools, and around 91.2% spending more than 10 h per week on social media. Most accessed the Internet via home WiFi or mobile data. Participants were also screened for their mental health status (See Supplementary Table S3).

**Figure 2:**
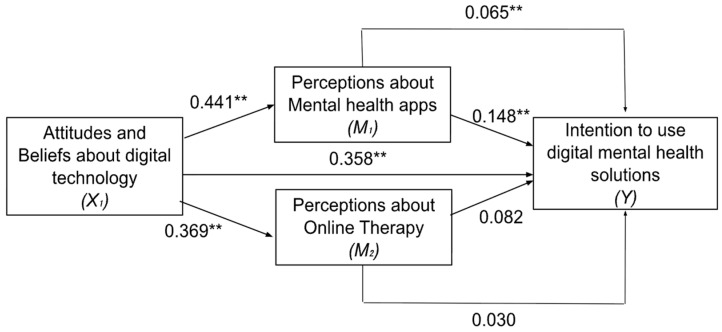
The final model. Note. ** Significant Effect (*p* < 0.05).

First, a confirmatory analysis (CFA) was conducted to evaluate the hypothesized four-factor measurement model, comprising Intention to use digital mental health solutions (Q1–Q4), Attitudes and beliefs about digital technology (Q5–Q14), Perceptions and beliefs about mental health apps (Q15–Q20), and Perceptions and beliefs about online therapy (Q21–Q27) (See Supplementary Note: Modification Indices). Overall results demonstrated an acceptable fit to the model (χ^2^(318) = 816, *p* < 0.001; CFI = 0.871; TLI = 0.858; RMSEA = 0.066; SRMR = 0.074). Most factor loadings were significant and within acceptable ranges, with only a few weaker items (Q12–Q14) and one marginally significant item (Q14). Composite reliability values were acceptable for three constructs (CR = 0.81–0.87) and slightly below the recommended threshold for Intention (CR = 0.66). Convergent validity was moderate (AVE = 0.35–0.49), and discriminant validity was supported via the Fornell–Larcker criterion, with moderate inter-factor correlations (r = 0.20–0.55). Overall, the measurement model demonstrated adequate reliability and validity, providing an acceptable fit for the subsequent mediation analysis. Figure 2 illustrates the final model, which summarizes the results of the mediation analysis, followed by a detailed description of the observed effects (Table 2).

As shown in Table 2, the findings revealed that attitudes towards digital technology had a significant, direct, positive effect on intention (β = 0.358, *p* < 0.001). Attitudes also significantly predicted perceptions of mental health apps (β = 0.441, *p* < 0.001) and online therapy (β = 0.369, *p* < 0.001). Furthermore, Perceptions of mental health apps had a strong direct effect on intention (β = 0.148, *p* = 0.004), whereas Perceptions of online therapy did not (β = 0.082, *p* = 0.101). Further Mediation analysis revealed that perceptions of mental health apps significantly mediated the relationship between digital attitudes and intention (indirect effect = 0.065, *p* = 0.006). In contrast, mediation via perceptions of online therapy was not significant (indirect effect = 0.030, *p* = 0.109). Moreover, the total indirect effect of digital attitudes through both mediators was significant (β = 0.095, *p* = 0.002), and the total effect on intention was strong and positive (β = 0.454, *p* < 0.001). These findings therefore suggest that positive attitudes toward digital technology increase the intention to use digital mental health solutions both directly and indirectly, primarily through enhanced beliefs about mental health apps.

## 4. Discussion

The present study explored the direct associative relationship between attitudes and beliefs about digital technology and the intention to use digital mental health solutions among SQU undergraduate students, while also examining whether perceptions of online therapy and mental health apps mediated that relationship. Our findings revealed that, overall, attitudes and beliefs about digital technology showed a positive relationship with intention and were positively associated with perceptions of both online therapy and mental health apps. However, mediation analysis revealed that only perceptions of mental health apps mediated this relationship, whereas perceptions of online therapy did not. Although the indirect effect of perceptions of online therapy was not statistically significant, this finding should be interpreted with caution. The confidence interval included small positive values close to zero, suggesting that a very small effect cannot be entirely ruled out. Therefore, rather than indicating a definitive absence of mediation, the result may be considered inconclusive. It is possible that this pathway has only a limited influence or that the study was not sufficiently sensitive to detect subtle effects. Future research with larger samples and stronger measurement approaches may help clarify this relationship further. The total indirect effect of both mediators was significant, revealing that service-specific perceptions (online therapy vs. mental health apps) partially explain how attitudes and beliefs towards digital tools drive intent to use digital mental health solutions.

The finding that attitudes and beliefs about digital technology was statistically associated with intention to use digital mental health solutions is consistent with current studies exploring attitudes for AI use in healthcare [21], and can be interpreted via the framework of the theory of planned behavior, suggesting that the more favorable technology is perceived in an individual, the more likely they will intend to utilize it [22]. Moreover, TPB also suggests that perceptions are largely driven by perceptions of the consequences of a behavior, all of which facilitate intention. For example, subjective norms play a vital role in determining the consequences of behavior [17]. Thus, individuals who perceive the subjective norm of using digital mental tools as acceptable may feel more in control, reflecting a higher intent to use such tools.

Moreover, the finding that positive perceptions of mental health apps strengthened intention to use digital mental health solutions could be associated with greater comfort in using personalized digital tools for support, compared to online therapy. To demonstrate, applications perceived as personal and secure may feel less socially risky than interacting with an online therapist, provided that previous studies have shown privacy concerns with using online tools for mental health [16]. Thus, engaging in real human interactions while subconsciously harboring such concerns could influence how users perceive online therapy as a useful tool [23]. Mobile mental health applications often facilitate autonomy and control over their engagement with psychological resources, which could lead to a more positive perception among individuals who value holding the privacy of their personal information without public disclosure.

Such findings are supported by multiple cross-cultural studies, specifically those that explored technology acceptance among patients seeking telemedicine services [24]. Their findings revealed that social influence and digital literacy were positively associated with intentions to use telemedicine apps. That is, if mobile applications were perceived as discreet and socially acceptable, individuals also perceived them as more useful than services that require live engagement with real mental health professionals. These findings underscore a crucial role of psychoeducation in promoting digital mental solutions as a resource before implementation.

Nevertheless, these results can be explained by the more specific constructs of the TPB, which could provide insights into how to navigate these newly innovative approaches to tackle mental health issues. According to this theory, human behavior is driven by several beliefs rather than one, which shape their perception of the extent to which they intend to use a certain behavior. These beliefs are shaped by considering the consequences of the behavior, the degree to which the individual is in control of the intended behavior, and the social pressure or norms related to the intended behavior [22]. In a collective society like Oman and other MENA regions, the likelihood of being perceived by another entity in a mental health application is often reduced compared to engagement with an online therapist, where the chances of being socially ostracized would be perceived as higher. In a society where social norms form a large part of one’s identity [25], a lower risk of being perceived negatively by others is an advantage for engagement.

Hence, our findings are partially in line with studies exploring the intention of digital mental health solutions in collectivist societies, which included various other contexts such as healthcare among medical and pharmacy students [18], within health education professionals [26], and of using digital mental health solutions for specific therapies such as CBT [27]. All together, the accessibility and 24/7 availability of digital mental health solutions are consistently valued within Omani society; hence, it is potentially their perception of its usefulness that mediates the extent to which they intend to use it for support.

While AI chatbots are discussed as a prominent emerging example of digital mental health technology, the constructs included in the SEM were measured at the broader level of mental health apps. Accordingly, the findings should be interpreted as reflecting general perceptions of digital mental health apps, rather than attitudes toward AI chatbots specifically.

Although the findings suggest positive intentions toward digital mental health solutions, they should be interpreted in light of the sample’s self-rated mental health profile. Most participants reported very good or excellent mental health, while only a small proportion reported poor mental health. Therefore, the findings may mainly reflect hypothetical intentions among students with relatively good perceived mental health, rather than actual help-seeking among students with current psychological distress. This limits direct clinical implementation and should be examined in future studies.

Overall, these findings highlight that positive attitudes toward digital technology may increase intention to use digital mental health solutions, particularly when linked to favorable perceptions of online therapy and mental health apps. They also suggest that digital readiness alone may be insufficient to drive adoption, as perceptions of digital tools are shaped by cultural, social, and identity-related factors.

### 4.1. Strengths and Limitations

This study has several strengths. It included a large sample of university students and used a previously validated questionnaire with acceptable overall reliability. Recruitment through multiple official and online channels helped reach a broad group of participants. The use of mediation analysis also provided a more nuanced understanding of how students’ attitudes toward digital technology may influence their intention to use digital mental health solutions, particularly through their perceptions of mental health apps.

However, several limitations should be considered. First, the cross-sectional design prevents causal inference. Second, all data were self-reported, which may introduce recall and social desirability biases.

The recruitment strategy may have introduced selection bias, as digitally engaged students may have been more likely to participate. The use of mandatory Google Forms responses resulted in complete data but may have introduced response bias. The response rate could not be calculated due to online distribution, and the single-university setting limits generalizability.

Although the proposed four-factor measurement model demonstrated an overall acceptable structure, several psychometric limitations should be considered when interpreting the mediation findings. Specifically, the CFI and TLI values fell below conventional thresholds for strong model fit, AVE values for all constructs were below the recommended criterion for optimal convergent validity, and the Intention construct showed marginal composite reliability. Collectively, these findings indicate that while the constructs were sufficiently distinguishable and generally reliable for exploratory mediation analysis, some latent variables, particularly Intention, may not have been measured with ideal precision. Consequently, the estimated indirect effects should be interpreted with caution, as measurement error and suboptimal convergent validity may attenuate or influence the strength and stability of the observed mediation pathways. Therefore, the current results should be viewed as preliminary but meaningful evidence supporting the hypothesized relationships, rather than definitive confirmation. Future research using refined instruments with stronger psychometric performance is warranted to validate and strengthen confidence in these mediation effects.

Finally, the SEM did not include potentially relevant covariates such as mental health status, prior psychological treatment, or digital literacy. Most participants also reported good, very good, or excellent mental health, which may have introduced a healthy participant bias. Therefore, the findings may primarily reflect hypothetical intention to use digital mental health tools among relatively healthy students, rather than the intentions or behaviors of students currently experiencing psychological distress. Future studies should use culturally validated measures, include relevant covariates, and recruit more diverse samples, including students with active mental health needs.

### 4.2. Clinical Implications

The present findings have crucial implications for implementing digital mental health tools clinically and at the community level. First, it highlights the role of psychoeducation on digital mental health tools before their introduction into healthcare services. This can be done through initial screening protocols that identify positive perceptions regarding digital mental health tools. Screening for perceptions of usefulness could help clinicians identify patients who are more likely to benefit from digital mental health services and optimize resource allocation by directing appropriate patients to digital support options while allocating traditional services to those who prefer face-to-face treatment. This approach may also help reduce the load on healthcare professionals and counseling services while diversifying accessible treatment for those seeking mental health care.

## 5. Conclusions

Overall, the present study explored the relationship between undergraduate students’ attitudes and beliefs about digital technology and their intent to use digital mental health solutions, and whether positive perceptions of mental health services, including online therapy and mental health apps, strengthen that relationship. Our findings suggest the role of a positive attitude toward digital technology in driving intention, with perceptions of mental health applications emerging as a potential psychological pathway. However, given the measurement limitations identified in this study, including the marginal composite reliability of the Intention construct, these findings should be interpreted cautiously. Subsequently, perceptions of online therapy did not significantly strengthen this relationship, although this should not be interpreted as definitive evidence that online therapy is less acceptable or less relevant. Rather, the difference between mental health applications and online therapy may reflect either a true difference in how students evaluate these services or measurement-related limitations. Mental health applications may represent a more accessible and potentially culturally acceptable pathway to mental health support, primarily due to their privacy and self-directed nature. Future research using more robustly validated measures is needed before drawing firm conclusions or making broad clinical recommendations based on these mediation findings. These findings inform technology acceptance policies by highlighting the importance of service-level perceptions in shaping behavioral intentions to use digital mental health services. Importantly, improving public perceptions of digital mental health applications is a crucial step in promoting digital access to valuable engagement with digital mental health support.

## Figures and Tables

**Table 1:** Sociodemographic information and personal characteristics.

Variable	*n* (%)
Gender	
Male	179 (49.7)
Female	181 (50.3)
Age, mean ± SD	21.24 ± 3.12
Nationality	
Omani	342 (95.0)
Non-Omani	18 (5.0)
Occupational Status	
Student	355 (98.6)
Non-Employed	2 (0.6)
Employed	3 (0.8)
Confidence in using smartphones, computers, and the Internet	
Not very confident	33 (9.2)
Slightly confident	47 (13.1)
Somewhat confident	90 (25.0)
Fairly confident	96 (26.7)
Very confident	94 (26.1)
Time spent on social media per week	
Don’t use social media	1 (0.3)
less than 5 h per week	31 (8.6)
10–20 h per week	150 (41.7)
20–30 h per week	114 (31.7)
30+ h a week	64 (17.8)
Uses a WiFi Fiber connection at home	288 (80.0)
Uses Mobile Data	282 (78.3)
Internet access on the university campus	242 (67.4)
Uses WiFi hotspots in public places	97 (26.9)
Money spent on data each month	
less than 5 OMR	96 (26.7)
5–10 OMR	93 (25.8)
10–15 OMR	80 (22.2)
15–30 OMR	20 (5.6)
Unlimited	71 (19.7)

**Table 2:** Direct, indirect, and total effects of Attitudes about digital technology, Perceptions of mental health apps, and online therapy, on intention to use digital mental health solutions according to the conceptual model. Std., standardized.

Model					95% Confidence Interval		*p*-Value
	Label	β (Std.)	SE	z	Lower	Upper	
Direct Effects							
Attitudes about digital technology (X) → Intention (Y)	c1	0.358	0.055	6.548	0.251	0.466	<0.001 *
Attitudes about digital technology (X) → Perceptions about mental health apps (M_1_)	a1	0.441	0.047	9.332	0.349	0.534	<0.001 *
Attitudes about digital technology (X) → Perceptions about online therapy (M_2_)	a2	0.369	0.049	7.543	0.273	0.465	<0.001 *
Perceptions about mental health apps (M_1_) → Intention (Y)	b1	0.148	0.052	2.862	0.047	0.249	0.004 *
Perceptions about online therapy (M_2_) → Intention (Y)	b2	0.082	0.050	1.640	−0.016	0.179	0.101
Indirect Effects							
Attitudes about digital technology (X) → Perceptions about mental health apps (M_1_) → Intention (Y)	a1 × b1	0.065	0.024	2.737	0.018	0.112	0.006 *
Attitudes about digital technology (X) → Perceptions about online therapy (M_2_) → Intention (Y)	a2 × b2	0.030	0.019	1.603	−0.007	0.067	0.109
Total Effects							
Attitudes about digital technology (X) → Intention (Y)	c1 + a1 × b1 + a2 × b2	0.454	0.047	9.663	0.362	0.546	<0.001 *
Total Effects (Indirect)							
Attitudes about digital technology (X) → Intention (Y)	a1 × b1 + a2 × b2	0.095	0.030	3.141	0.036	0.155	0.002 *

Note. * Significant Effect.

## Data Availability

The original contributions presented in this study are included in the article/Supplementary Materials. Further inquiries can be directed to the corresponding author.

## Supplementary Materials

The following supporting information can be downloaded at: https://www.mdpi.com/article/10.3390/ijerph23060719/s1. Table S1: Perceptions and beliefs about mental health apps; Table S2: Mental Health App Priorities Among University Students; Table S3: Mental health and treatment history. Supplementary Note: Modification Indices (MI).
