# Supplementary material for: University Students’ Perceptions and Intentions to Use Digital Mental Health Services Including Online Therapy and Mental Health Apps: A Cross-Sectional Study
Source: Int J Environ Res Public Health. 2026 May 28;23(6):719. doi: 10.3390/ijerph23060719 (PMC13300285; doi:10.3390/ijerph23060719)
Supplement: Supplementary file 1 [file ijerph-23-00719-s001.zip › ijerph-4300161-supplementary.pdf]

Table S1. Perceptions and beliefs about mental health apps.

Variable	Not at all important n (%)	Not very important n (%)	Somewhat important n (%)	Very important n (%)
Data protection and privacy	16 (4.4)	18 (5.0)	41 (11.4)	285 (79.2)
Recommendations of other students	21 (5.8)	67 (18.6)	177 (49.2)	95 (26.4)
Recommendations by a mental health professional	12 (3.3)	30 (8.3)	104 (28.9)	214 (59.4)
Apps developed specifically for students	21 (5.8)	85 (23.6)	143 (39.7)	111 (30.8)
Apps developed by South Africans	52 (14.4)	91 (25.3)	117 (32.5)	100 (27.8)
Knowing that the app was approved by the Health Professions Council or the government department of health	16 (4.4)	31 (8.6)	94 (26.1)	219 (60.8)

Table S2. Mental Health App Priorities Among University Students.

Variable	n (%)
If mental health apps were going to be offered to university students, which of the following problems should they focus on?	
Stress reduction	292(81.3)
Depression	246(68.5)
Anxiety	258(71.9)
Procrastination	179 (49.9)
Time management	249 (69.4)
Study Skills	217 (60.4)
Attention and Concentration Improvement	227 (63.2)
Mindfulness	135 (37.6)
Help to stop smoking	85 (23.7)
Help to reduce alcohol consumption	66 (18.4)
Help to reduce drug use	70 (19.5)
Wearable electronics to track physical metrics, consumer wearables	33 (9.2)

Table S3. Mental health and treatment history.

Variable	n (%)
Current mental health status	
Poor	8 (2.2)
Fair	32 (8.9)
Good	96 (26.7)
Very good	137 (38.1)
Excellent	87 (24.2)
Received psychological counseling	44 (12.2)
Received medication for mental health	26 (7.2)
Received any treatment/counseling	32 (8.9)
